# Low-affinity FcγR interactions can decide the fate of novel human IgG-sensitised red blood cells and platelets

**DOI:** 10.1002/eji.201343825

**Published:** 2014-02-16

**Authors:** Kathryn L Armour, Cheryl S Smith, Craig P Turner, Christopher M Kirton, Anthony M Wilkes, Andrew G Hadley, Cedric Ghevaert, Lorna M Williamson, Michael R Clark

**Affiliations:** 1 Department of Pathology, University of CambridgeCambridge, UK; 2 Bristol Institute for Transfusion SciencesBristol, UK; 3 NHS Blood and TransplantCambridge, UK; 4 Department of Haematology, University of CambridgeCambridge, UK

**Keywords:** Blocking antibody, Fc engineering, IgG effector function, Low-affinity Fc receptors

## Abstract

G1Δnab is a mutant human IgG1 constant region with a lower ability to interact with FcγR than the natural IgG constant regions. Radiolabelled RBCs and platelets sensitised with specific G1Δnab Abs were cleared more slowly from human circulation than IgG1-sensitised counterparts. However, non-destructive splenic retention of G1Δnab-coated RBCs required investigation and plasma radioactivities now suggest this also occurred for platelets sensitised with an IgG1/G1Δnab mixture. In vitro assays with human cells showed that G1Δnab-sensitised RBCs did not cause FcγRI-mediated monocyte activation, FcγRIIIa-mediated antibody-dependent cell-mediated cytotoxicity (ADCC) or macrophage phagocytosis although they did adhere to macrophages. Thus, FcγRII was implicated in the adhesion despite the Δnab mutation reducing the already low-affinity binding to this receptor class. Additional contacts via P-selectin enhance the interaction of sensitised platelets with monocytes and this system provided evidence of FcγRII-dependent activation by G1Δnab.

These results emphasise the physiological relevance of low-affinity interactions: It appears that FcγRII interactions of G1Δnab allowed splenic retention of G1Δnab-coated RBCs with inhibitory FcγRIIb binding preventing RBC destruction and that FcγRIIb engagement by G1Δnab on IgG1/G1Δnab-sensitised platelets overcame activation by IgG1. Considering therapeutic blocking Abs, G1Δnab offers lower FcγR binding and a greater bias towards inhibition than IgG2 and IgG4 constant regions.

## Introduction

We have been developing an inert constant region for use in therapeutic Abs with blocking functions. We observed in human volunteers that cells coated with an Ab containing this constant region were unexpectedly sequestered in the spleen. We wished to investigate whether the residual, low-affinity interactions of the Fc might be responsible.

We designed the inert Fc to lack cytotoxic activity but to retain the FcRn binding crucial for long half-life and placental transport. To avoid the creation of new immunogenic epitopes, we incorporated motifs from human IgG2 (residues 233–236) and IgG4 (327, 330 and 331) into an IgG1 constant region 1. When combined with human anti-RhD variable regions (Fog-1 2), this constant region, G1Δab, showed minimal binding to FcγRI and FcγRIII such that Fog-1 G1Δab-sensitised RBCs did not activate monocytes and were not lysed by NK antibody-dependent cell-mediated cytotoxicity (ADCC). Moreover, Fog-1 G1Δab was able to inhibit the triggering of these activities by either Fog-1 IgG1 wild-type (WT) Ab (Fog-1 G1) or clinical anti-RhD sera 1,3. Since binding to the low-affinity FcγRII molecules was also reduced 4, G1Δab appeared to be a good candidate for an inert constant region and was further modified to G1Δnab to eliminate allotypic residues 5.

In a volunteer study, aliquots of autologous RBCs were labelled with different radionuclides before being coated with either Fog-1 G1 or G1Δnab Ab 5. After re-injection, there was complete, irreversible clearance of G1-coated cells, with accumulation in the spleen and liver and the appearance of radiolabel in plasma. The clearance of cells coated with Fog-1 G1Δnab was significantly slower and, surprisingly, was incomplete and transient with blood cell counts rising again after 3–4 h. Scans showed that G1Δnab-coated cells accumulated in the spleen but no radiolabel was detected in the plasma. These findings suggest that G1Δnab-sensitised cells were not destroyed but showed exaggerated pooling within the spleen. The net increase in circulating, labelled cells at later time points possibly occurred due to elution of G1Δnab from the RhD antigen (Ag).

A second G1Δnab volunteer study was related to a possible blocking Ab treatment for the condition feto-maternal alloimmune thrombocytopenia, which is due to transplacental passage of maternal anti-human platelet Ag (HPA) Abs, usually anti-HPA-1a. It causes severe fetal thrombocytopenia in 1 in 1200 unselected pregnancies and intrauterine death or intracerebral haemorrhage can result 6. The current antenatal therapy of high-dose intravenous immunoglobulin is unsatisfactory. An inert HPA-1a-specific Ab could be administered to the mother to cross the placenta and block the interaction of the maternal alloantibodies with HPA-1a on fetal platelets without causing platelet destruction itself. An anti-HPA-1a version of G1Δnab was produced with highly specific variable regions (B2 7,8). B2 G1Δnab reduced the monocyte chemiluminescence (CL) response to platelets that were sensitised with B2 IgG1 WT Ab (B2 G1) or with a range of maternal sera containing HPA-1a Abs 9. The intravascular survival of unsensitised, autologous platelets was compared with the survival of those sensitised with B2 G1, B2 G1Δnab or a mixture of the two Abs 10. Platelets sensitised with G1 (P-G1) were completely cleared from the circulation in 2 h, whilst platelets sensitised with G1Δnab (P-G1Δnab) showed the same survival as unsensitised platelets (P). Platelets that were sensitised with a combination of B2 G1 and B2 G1Δnab (P-G1/G1Δnab) were cleared from the circulation similarly to P-G1 but with an improved survival of two- to threefold. We have now analysed the data for the levels of radiolabel appearing in the plasma to see how clearance relates to destruction for each platelet type.

We have investigated the molecular basis for our in vivo observations by assaying Fog-1 and B2 G1Δnab in FcγR binding and functional assays. We mimicked the situation in the spleen by looking at the interaction between Fog-1-sensitised RBCs and macrophages. It is difficult to attribute low-affinity interactions of IgGs to particular FcγR in functional assays but it is known that when human monocytes are activated by sensitised platelets, IgG-FcγR interactions are enhanced by additional association through P-selectin 11. Thus, this was an ideal system for studying low-affinity FcγR binding. Assays were made more informative by including anti-HPA-1a Abs with different FcγR-binding profiles alongside B2 G1 and B2 G1Δnab. These were an IgG2 WT molecule (B2 G2) and B2 G1Δnac. The G1Δnac constant region is identical to G1Δnab except that it includes the IgG1 residue G236, which is absent in IgG2 and G1Δnab. Previously, G1Δab and G1Δac molecules were shown to be similarly non-destructive overall but to exhibit low levels of activity in different assays 1,3,4. In this way, we hoped to discover which interactions of the G1Δnab constant region were relevant to the Abs’ behaviour in vivo.

## Results

### Platelet survival study: Analysis of plasma-associated radioactivity

**Figure 1:**
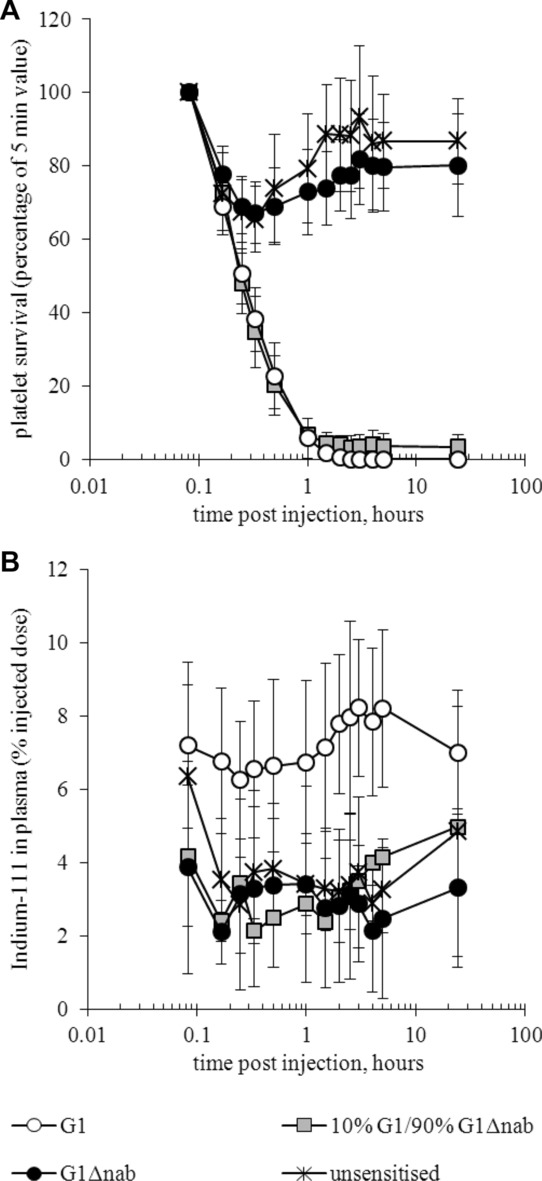
Platelet survival study: intravascular survival and radioactivity associated with the plasma for selected platelet samples. (A) Intravascular platelet survival is calculated by expressing the ^111^In radioactivity of the cellular fraction of each blood sample as a percentage of the 5 min value for that volunteer. (B) The plasma-associated ^111^In radioactivity levels are given as a percentage of the ^111^In activity injected. Data relate to ^111^In-labelled samples of unsensitised platelets, P-G1, P-G1Δnab and P-G1/G1Δnab in three, five, four and two volunteers, respectively. For unsensitised platelets, only data from volunteers who received G1Δnab-coated, ^51^Cr-labelled platelets alongside are included as, when G1 was present on the other platelets, higher levels of plasma ^111^In were seen, presumably due to IgG exchange in the pre-injection mixture. Thus, data are restricted to the ^111^In-labelled samples of volunteers 1–7, 9 and 13–18 (detailed in 10). The curves for each type of platelet represent the mean ± SD of the activities in the different individuals or, for P-G1/G1Δnab, the range of the activities in the two individuals.

Each volunteer in the platelet survival study received two samples of autologous, HPA-1a1b platelets that had been left unsensitised or sensitised at saturating concentrations of B2 Ab (0.13 mg/mL) and then labelled with different radionuclides 10. The previous report focussed on the survival curves generated from the radioactivity in the cellular fractions of the blood samples 10. Platelet destruction can be inferred from radioactivity appearing in the plasma but only for platelets radiolabelled with ^111^In since ^51^Cr elutes too rapidly. This limits the number of data sets available for each type of platelet: unsensitised (*n* = 3), P-G1 (*n* = 5), P-G1Δnab (*n* = 4) or P-G1/G1Δnab (10% B2 G1/90% B2 G1Δnab, *n* = 2). Figure1 compares the plasma-associated ^111^In radioactivity levels measured for the four types of platelets and shows the corresponding platelet survival curves when data are restricted to these platelet samples. The graphs are limited to the first 24 h after injection because B2 Abs redistribute to the whole platelet population by this time point 10. Large error bars result from donor variation and the small group sizes mean statistics cannot be applied but there was a higher level of plasma ^111^In activity associated with P-G1 than for the other types of platelets. The result is particularly striking for P-G1/G1Δnab, given that the survival curves for these platelets and P-G1 are similar. In fact, one of the volunteers receiving the ^111^In-labelled P-G1/G1Δnab had significantly higher HPA-1a levels on their platelets than all other volunteers (UPN 18; see table 1 of 10). These P-G1/G1Δnab were cleared more quickly than all other samples of P-G1/G1Δnab but this was not accompanied by increased levels of ^111^In in the plasma.

### Binding of anti-RhD and anti-HPA-1a Abs to FcγR

**Figure 2:**
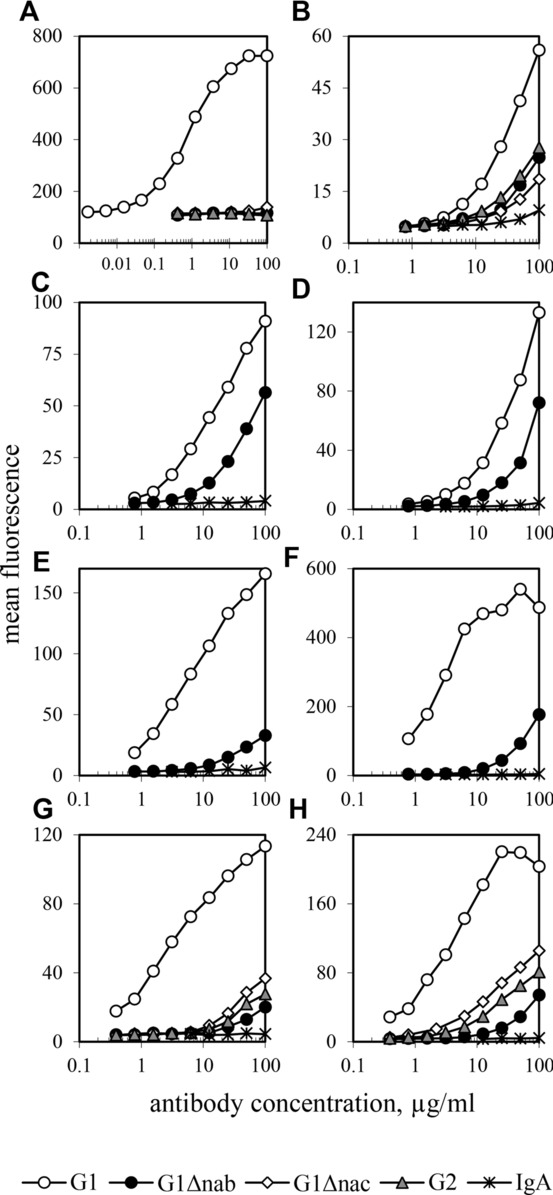
Binding interactions of Fog-1 and B2 IgG variants with human FcγR. (A) Binding of monomeric B2 IgGs was measured for the high-affinity FcγRI using the B2KA cell line and flow cytometry. (B–H) Binding of (B, G, H) pre-complexed B2 or (C–F) Fog-1 IgGs was measured using CHO cell lines expressing FcγRIIa of allotypes (B) 131R and (C) 131H, (D) FcγRIIb and FcγRIIIa of allotypes (E, G) 158F and (F, H) 158 V. The level of background binding is given by the negative control Ab, IgA,κ or IgA,λ as appropriate. Graphs show mean fluorescence of ≥12 000 cells at each Ab concentration and are typical of the results obtained in at least three experiments with each receptor.

To investigate the basis for the removal of the G1Δnab-sensitised RBCs and platelets from the circulation, we used transfected cell lines, each expressing a single human FcγR, to assess the level of interaction of G1Δnab Abs in comparison with the WT IgG1 controls. For the anti-HPA-1a Abs, we also included the WT IgG2 (B2 G2) and the mutant B2 G1Δnac. Binding of monomeric IgG to the high affinity FcγRI was measured for the Fog-1 (not shown) and B2 Abs (Fig.2A). G1 bound strongly whereas no binding of G2 or G1Δnab was detected at concentrations ≤100 μg/mL. G1Δnac showed a small degree of binding at ≥30 μg/mL.

For the lower affinity receptors of the FcγRII and III classes, the binding of pre-complexed IgG was measured so that the avidity effect would allow low levels of interaction to be visualised. For FcγRIIa, of allotypes 131R and 131H, and FcγRIIb, Fog-1 G1Δnab bound three- to eightfold less strongly than Fog-1 G1 but substantially more than IgA negative control (not shown and Fig.2C and D). With the B2 Abs, G1Δnab bound more strongly than G1Δnac to FcγRIIa, of allotypes 131R (Fig.2B) and 131H (not shown). These two mutants showed approximately equal binding to FcγRIIb (not shown). For both Fog-1 and B2 Abs, G1Δnab binding to FcγRIIIa was above that of the IgA negative control but was approximately 100-fold (158F allotype) or 50-fold (158V allotype) lower than G1 binding (Fig.2E–H). In the B2 Ab set, G1Δnac and G2 both bound more strongly than G1Δnab to FcγRIIIa (Fig.2G and H). For FcγRIIIb, of NA1 and NA2 allotypes, only Fog-1 G1 or B2 G1 complex binding could be detected at concentrations ≤100 μg/mL (not shown).

### Functional assays of responses to Fog-1-sensitised RBCs

**Figure 3:**
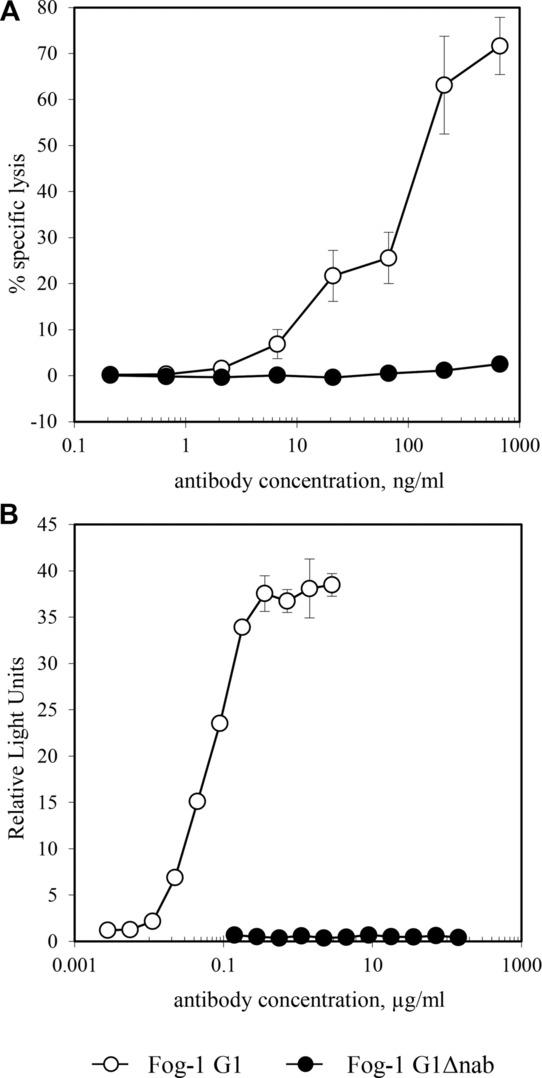
Functional responses to RBCs sensitised with Fog-1 G1 and G1Δnab Abs. (A) The specific lysis of sensitised RBCs by NK-cell-mediated ADCC is presented as mean ± SD of triplicate samples. This experiment used effector cells pooled from six donors but similar results were obtained in four experiments with individual donors of PBMCs. (B) The mean CL response of monocytes to sensitised RBCs is plotted, with the error bars indicating the range of the duplicate samples. Data shown are from one experiment representative of three experiments performed.

Saturation of RBC RhD sites was achieved at coating concentrations of 20 μg/mL and 50% saturation at approximately 0.4 μg/mL for all Fog-1 Abs (not shown). Measurement of NK-cell-mediated ADCC of Fog-1 IgG-sensitised RBCs showed G1 to be highly active at sub-saturating concentrations whilst any lysis caused by G1Δnab was at background levels (Fig.3A). Fog-1 G1-sensitised RBCs efficiently activated monocytes, as seen by their CL response, whereas G1Δnab-sensitised RBCs did not cause activation even when the RhD sites were saturated with Ab (Fig.3B).

**Figure 4:**
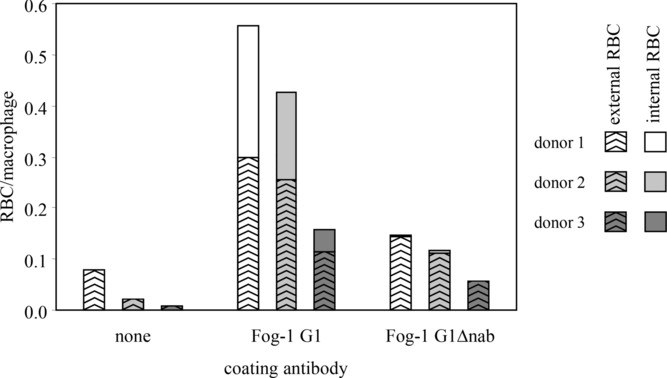
Interactions of Fog-1 IgG-sensitised RBCs with macrophages. The numbers of adherent and phagocytosed RBCs per macrophage were determined for unsensitised RBCs and RBCs sensitised with saturating concentrations of Fog-1 G1 or G1Δnab. Results for macrophages from three different donors are shown.

RBCs, whose RhD sites had been saturated with Fog-1 Ab, were incubated with macrophages to assess their ability to interact. The number of G1Δnab-coated RBCs associated with the macrophages was significantly greater than for unsensitised cells (*p* < 0.05, paired Student's *t*-test) and, for each donor, amounted to approximately 30% of the numbers of associated G1-sensitised cells (Fig.4). However, very few G1Δnab cells were within the macrophages and the proportion of macrophage-associated G1-sensitised RBCs that had been internalised was significantly larger (*p* < 0.05, paired Student's *t*-test). Furthermore, the macrophages incubated with the unsensitised and G1Δnab-sensitised RBCs retained a contracted morphology whilst those incubated with the G1-coated cells had spread.

### Monocyte CL responses to HPA-1a Abs

**Figure 5:**
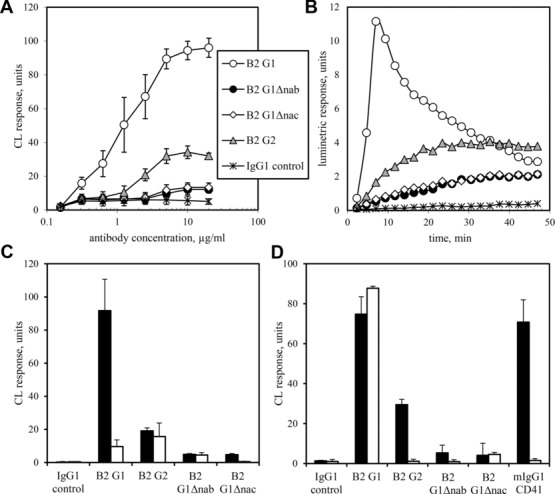
The CL responses of monocytes to HPA-1a/1b platelets sensitised with anti-HPA-1a IgGs. (A) The magnitude and (B) kinetics of the monocyte CL response to HPA-1a/1b platelets sensitised with B2 Ab or a human IgG1 isotype control were measured. (C, D) The effects on the magnitude of the CL response of blocking (C) FcγRI with monomeric murine IgG2a or (D) FcγRII with Fab fragments of mAb IV.3 were determined. Monocytes were incubated with HBSS (black bars) or 20 μg/mL blocking agent (white bars), prior to the addition of platelets sensitised with 10 μg/mL IgG1 isotype control, 1 μg/mL B2 G1, 5 μg/mL B2 G2, 10 μg/mL B2 G1Δnab, 10 μg/mL B2 G1Δnac or 5 μg/mL CD41 (murine IgG1). Throughout, magnitudes are shown as mean + or ± SD from three experiments, each carried out in duplicate. The kinetic curves are shown for platelets sensitised with 20 μg/mL each IgG in a representative experiment.

HPA 1a/1b platelets were used to match the genotype of platelets in the survival study. The four B2 Abs gave identical platelet binding curves, with saturation being approached at 5–10 μg/mL (not shown). The monocyte CL response to P-G1 or platelets sensitised with B2 G2 (P-G2) was dependent on the degree of sensitisation and was maximal at 10 μg/mL Ab (Fig.5A). The response to P-G2 was approximately 30% of that to P-G1 at each Ab concentration. CL responses to P-G1Δnab and platelets sensitised with G1Δnac (P-G1Δnac) were similar at 12–13% of P-G1 values (*p* < 0.0001) across the concentration range but were greater than those observed to the control Ab at concentrations ≥5 μg/mL (*p* < 0.0001). No CL response was obtained when HPA-1b/1b platelets were used (not shown). When viewed as CL signal against time, the response to P-G1 is characterised by a rapid rise in signal that peaks before 10 min whilst the other B2 Abs cause a slow signal increase that approaches a plateau at 45 min (Fig.5B). As the coating concentration of B2 G1 is decreased, the rapid rise in CL signal is lost such that a curve for 1 μg/mL G1 resembles that for 10 μg/mL G2 (not shown). The residual CL response to P-G1Δnab or P-G1Δnac was not due to the mutated Abs causing platelet activation since the P-selectin expression of platelets sensitised with 100 μg/mL each B2 IgG was not significantly different from platelets incubated with an IgG1 isotype control (MFI 0.7 ± 0.8 units), whereas thrombin-stimulated platelets gave much higher expression (MFI 16.2 ± 1.3 units).

The above experiments measured the response of monocytes to pre-sensitised platelets. A more rapid response was seen when HPA-1a Abs were added to pre-adhered monocytes and platelets. The CL signal peaked before the first time point (not shown) and the calculated CL response was greater in magnitude. Using concentrations of each B2 Ab that elicited sub-maximal CL responses, the increase attributable to pre-adhesion amounted to twofold for 1 μg/mL G1, threefold for 5 μg/mL G2 and seven- to eightfold for 10 μg/mL G1Δnab or G1Δnac. There was no response to IgG1 isotype control or when using HPA-1b/1b platelets (not shown).

### Characterisation of the FcγR involved in the CL responses to HPA-1a Abs

Monocytes were pre-incubated with blocking agents to inhibit IgG binding to FcγRI or FcγRII. When 20 μg/mL monomeric murine IgG_2a_ Ab was used to block FcγRI, the CL responses to P-G1 and P-G1Δnac were reduced by 90% whereas CL responses to P-G1Δnab and P-G2 were only reduced by 9 and 18%, respectively (Fig.5C).

Fab fragments of the anti-FcγRII mAb IV.3 were used to block binding to FcγRIIa. IV.3 whole antibody staining of FcγR-expressing cell lines has shown it to have 1000-fold greater affinity for FcγRIIa than FcγRIIb (not shown) so use of IV.3 Fab will have resulted in little FcγRIIb blocking. Donors homozygous for the 131R allotype of FcγRIIa, which binds murine IgG_1_, were used to enable demonstration of efficient blocking of FcγRIIa by IV.3 Fab: the CL response to a murine IgG_1_ CD41Ab was inhibited by 98%. Responses to P-G2 and P-G1Δnab were reduced by more than 80% to control levels whereas responses to P-G1 and P-G1Δnac were not inhibited (Fig.5D). Indeed, responses to P-G1 tended to be enhanced.

## Discussion

Our human volunteer studies have now revealed two instances of non-destructive sequestration that might be explained by low-affinity IgG-FcγR interactions. Previously, in the RBC survival study, imaging showed that Fog-1 G1Δnab-coated cells were retained in the spleen but, since there was no release of radiolabel into the plasma, any interaction with splenic FcγR did not drive their destruction 5. Now, evaluation of the ^111^In activity levels in the plasma fractions from the platelet survival study 10 has shown low activities for unsensitised platelets, P-G1Δnab and, remarkably, P-G1/G1Δnab whereas higher activities reflected the rapid rate of P-G1 destruction. The shape of the P-G1/G1Δnab survival curves had suggested platelet destruction albeit at a slower rate than for G1-coated platelets: the calculated survival of P-G1/G1Δnab was 58 ± 12 min against 18 ± 5 min for P-G1 for all data sets 10. Plasma activity levels suggest that the loss of circulating P-G1/G1Δnab was due to sequestration rather than destruction. Thus, the behaviour of these platelets paralleled that of the G1Δnab-coated cells in the RBC study and may have been due to prolonged splenic pooling. The proposed G1Δnab treatment of thrombocytopenic fetuses should result in all platelets being coated with a mixture of active and inactive IgGs so, assuming a limited capacity for splenic retention, most platelets would remain in circulation.

The interaction between Fog-1-sensitised RBCs and macrophages was investigated in vitro as a model for the interactions between G1Δnab Abs and splenic FcγR and produced two important results. Firstly, although the numbers of G1Δnab-sensitised RBCs associated with the macrophages were threefold lower than for G1-sensitised RBCs, they were significantly higher than for unsensitised RBCs. This suggests that the interactions of G1Δnab with one or more macrophage FcγRs, namely FcγRI, FcγRIIa, FcγRIIb and FcγRIIIa 12, are strong enough to promote association. G1Δnab-sensitised RBCs did not trigger monocyte activation or ADCC and these activities are known to be FcγRI- and FcγRIIIa-mediated, respectively 3,11. Thus, it is most likely that G1Δnab-sensitised RBCs adhered to macrophages via interactions with FcγRIIa or FcγRIIb. The second observation was that a much lower proportion of macrophage-associated RBCs were internalised for Fog-1 G1Δnab compared to Fog-1 G1. Due to inter-donor variation, perhaps resulting from differences in levels of FcγR expression or from receptor polymorphisms, G1Δnab-sensitised RBCs associated with donor 1 macrophages to a similar extent as G1-sensitised RBCs with donor 3 macrophages. Even in these circumstances, greater numbers of the G1 cells were internalised. Perhaps G1Δnab-sensitised RBCs did not trigger phagocytosis because their interactions with activating FcγR were insufficient to overcome the inhibitory effect of cross-linking FcγRIIb.

The numbers of G1Δnab-sensitised RBCs adhering to macrophages were too small to allow easy investigation of the types of FcγR involved. Monocytes also express FcγRI, FcγRIIa, FcγRIIb and, sometimes, FcγRIIIa 12 and their activation by Ab-coated platelets is a more sensitive system as it is partially mediated by the interaction between platelet P-selectin and P-selectin glycoprotein ligand-1 on monocytes 11. This allowed the effects of blocking the different FcγR to be examined but, since B2 G1Δnab exhibits only low-affinity FcγR binding, it was important to show that a specific interaction was being blocked. As a control, we used B2 G1Δnac, an IgG that also showed only low-affinity FcγR binding but with a distinct receptor profile. In addition to the B2 Ab binding experiments here, FcγR binding of G1Δab and G1Δac constant regions has been compared in the context of Fog-1 and CAMPATH variable regions without the null allotype mutations 1,3,4. The hierarchy of binding to each receptor was retained whatever the setting of the mutations. Binding to FcγRI of Δab-containing Abs was at background levels like IgG2 whereas Δac mutants retained slight binding at 100 μg/mL. It was with FcγRII receptors that the mutations had the least effect on binding; in particular, binding to the inhibitory receptor, FcγRIIb, was only reduced by three- to fourfold. The hierarchies were G1 > G2 > G1Δab > G1Δac for FcγRIIa of the 131R allotype, G2 ≥ G1 > G1Δab > G1Δac for FcγRIIa 131H and G1 > G2 > G1Δab ≈ G1Δac for FcγRIIb. For FcγRIIIa, the order was G1 >> G1Δac > G2 > G1Δab whereas, of these Abs, only G1 showed measurable binding to FcγRIIIb. The G1Δnab and G1Δnac constant regions differ only by the absence or presence of G236. It has been suggested that the flexibility given by G236 and G237, as in the IgG1 lower hinge, is required for binding to FcγRI and FcγRIII due to the tightness of the fit but that the equivalent contact residues of FcγRII are smaller and allow binding of the IgG2 lower hinge without G236 13. This might explain the binding preferences of G1Δnab and G1Δnac.

The tethering via P-selectin allowed P-G1Δnab and P-G2 to activate monocytes at 12–13% and 30% of the P-G1 level whereas G1Δnab- or G2-sensitised RBCs did not elicit any response from monocytes here and previously 1. There was no increase in P-selectin upon Ab binding so the monocyte responses to B2 G1Δnab, G1Δnac and G2 Abs were not artefacts caused by platelet activation. However, the CL response to B2 G1 showed different kinetics to the responses to the other B2 Abs. The slow CL responses to P-G1Δnab, P-G1Δnac or P-G2 may have been due to platelets first adhering to monocytes through P-selectin and then causing activation through low-affinity interactions with FcγR. The fast response to P-G1 reflected the strong binding of IgG1 to FcγRI that negated the need for prior P-selectin interactions except at low Ab density. This fits with the observations that unsensitised platelets can adhere to monocytes and that the CL response upon subsequent addition of Ab is faster and greater than when sensitised platelets are added to monocytes 11. This pre-adhesion enhancement operated for all four B2 variants. The association of platelets with monocytes appears to be the slowest event in the monocyte activation. Following pre-adhesion, the close proximity of the platelet Ag and monocyte FcγR presumably allows higher avidity Ab binding and offsets the time taken for the Abs to diffuse to the binding sites.

The binding profiles of B2 G1Δnab and G1Δnac were reflected in the pattern of FcγR utilisation in the monocyte activation experiments. Despite the low activity exhibited by the mutant IgGs, the FcγRI and FcγRII blocking agents each eliminated CL due to one mutant but not the other, which indicated that specific FcγR-mediated events were being inhibited. Importantly, blocking FcγRIIa abolished the response due to G2 and G1Δnab, which supports our suggestion that it is the interactions of G1Δnab with FcγRII that are biologically relevant. Blocking FcγRI inhibited G1 and G1Δnac activity although, as previously 11, the response to G1 could not be eliminated entirely. Presumably, this is because the Ab could act through FcγRII in the presence of P-selectin tethering. Conversely, blocking FcγRII did not reduce the response due to G1 and G1Δnac and tended to enhance responses to P-G1, as was previously reported for RBCs and platelets sensitised with IgG1 11. The selectivity of IV.3 for FcγRIIa over FcγRIIb means the enhancement was not caused by blockade of the inhibitory receptor but perhaps by forcing G1 to interact with FcγRI rather than, less productively, with FcγRIIa.

We have shown that Fog-1 G1Δnab-sensitised RBCs can adhere to macrophages without this resulting in phagocytosis and that the major interactions of G1Δnab Ab are with FcγRII. In vivo, the interactions used by macrophages to survey RBCs for signs of aging 14, possibly enhanced by ex vivo alterations to the RBCs, might augment the modest Ab binding to FcγRIIa and FcγRIIb. Nevertheless, a large proportion of the Fog-1 G1Δnab-coated RBCs retained by splenic macrophages was returned to the circulation rather than undergoing phagocytosis. In contrast, there was no evidence for Ab-mediated splenic retention of platelets sensitised with G1Δnab alone. Although levels of circulating P-G1Δnab fell before rising to a plateau at 2 h post-injection, this behaviour was observed for unsensitised platelets concurrently and previously 10,15. Platelet distribution may have been temporarily affected by partial activation during ex vivo handling, which was substantiated by the small increases in P-selectin levels observed for all platelet types 10. When labelled platelets were infused as part of autologous platelet transfusions, intravascular levels fell for the first hour whilst activity over the spleen increased 15. A dynamic equilibrium of labelled platelet distribution between organ pools and circulation was reached after 2 h.

In the platelet survival study, it was the platelets sensitised with B2 G1Δnab and G1, rather than G1Δnab alone, for which there was evidence of Ab-mediated retention in the spleen. In this case, G1 and P-selectin would be acting to promote association with splenic macrophages, which would allow G1Δnab to bind to FcγRII. The balance between the interactions with the activating FcγR and the inhibitory FcγRIIb would then determine the fate of the captured platelets. It appears that the alteration in bias of the G1Δnab constant region away from activatory receptor binding is sufficient to prevent destruction of these platelets.

We have seen other demonstrations of tethering through a high avidity interaction (as through P-selectin) giving significance to low-affinity interactions that are not themselves strong enough to promote cell-to-cell interactions. When testing the ability of Fog-1 IgGs to inhibit G1-mediated monocyte CL, we saw that G2 was not as efficient an inhibitor as some mutant IgGs despite the inability of G2-coated RBCs to stimulate CL activity 1. The reduced capacity of G2 to inhibit was eliminated by blockade of FcγRII (AGH unpublished data). Similarly, an IgG2 Ab was less effective than a mutant IgG2 in inhibiting the capture of neutrophils by IgG1 even though IgG2 was unable capture neutrophils when used alone 16. The potential for low-affinity interactions to amplify the effects of higher affinity interactions has implications for the roles of IgG2 and IgG4 in vivo and, in particular, hinders the use of these subclasses as ‘inert’ constant regions for therapeutic blocking Abs. The shift in activation/inhibition bias exhibited by G1Δnab, coupled with its lower FcγR binding overall, means it should be considered as an alternative to IgG2 or IgG4 when selecting a constant region for blocking Abs.

## Materials and methods

### Ab production

Production and characterisation of recombinant IgG1 and mutant G1Δab forms of Fog-1 have been described 1,3,4. Fog-1 G1Δnab was produced by removing the G1m(1,17) allotypic residues from G1Δab, without effect on its properties 5.

Generation of B2 G1, a human IgG1,λ version of an anti-HPA-1a single-chain Fv, and B2 G1Δnab have been described 8,9. B2 IgG2 and G1Δnac heavy chain vectors were constructed by exchange of restriction fragments between existing vectors 1,8,9 and were each cotransfected with the B2 λ-chain expression vector 8 to produce B2 G2 and B2 G1Δnac Abs.

### Platelet survival study: Analysis of plasma-associated radioactivity

The determination of the in vivo survival of platelets, which were unsensitised or sensitised with B2 G1, B2 G1Δnab or a mixture of these two Abs, has been described 10. Local Ethical Committee approval and informed consent of all subjects were obtained. For each volunteer, two samples of autologous platelets received a different sensitisation and were radiolabelled with ^51^Cr or ^111^In before re-infusion. Adjusted counts for the cellular fractions of samples taken post-injection were used to generate platelet survival curves. Here, the data for the radioactivity associated with the plasma have been analysed. This is only informative for platelets labelled with ^111^In since ^51^Cr shows a high elution rate. Adjusted ^111^In counts for the plasma fractions were expressed as a percentage of the injected dose and combined for volunteers receiving the same Ab combinations.

### Cell lines bearing human FcγR

Cell lines transfected with cDNA expression vector constructs to express single human FcγR have been variously obtained. For FcγRI, the cell line was B2KA (S. Gorman and G. Hale, unpublished) and CHO cells expressing FcγRIIIb of allotypes NA1 and NA2 17 were kindly provided by J. Bux. FcγRIIIa of allotypes 158F and 158V were expressed as GPI-anchored receptors in CHO 18.

FcγRIIa of allotypes 131R and 131H and FcγRIIb were expressed in CHO cells as transmembrane proteins. Briefly, cDNA was synthesised from human PBMC RNA using specific primers and amplified by nested PCR to yield HindIII – XbaI DNAs, which comprised the whole receptor coding region including signal sequence and cytoplasmic domain. cDNAs encoding FcγRIIa 131H and FcγRIIb were obtained by mutation of the highly homologous FcγRIIa 131R and FcγRIIc DNAs, respectively. cDNAs were inserted into pcDNA3.1/Hygro(+) (Invitrogen, Paisley, UK), transfected into CHO cells and receptor-expressing clones isolated as described 18.

### Measurement of binding to FcγR transfectants

Binding of monomeric IgG to B2KA cells expressing FcγRI was measured as previously described 1 except that, for the B2 Abs, 80 μg/mL biotin-conjugated goat anti-human λ-chain Abs (Sigma, Poole, UK) were used as the first detection reagent.

Binding to FcγRII and III receptors was measured by pre-complexing the Abs with equimolar amounts of F(ab’)_2_ fragments, which recognised the light chain 4: goat F(ab’)_2_ anti-human κ (Rockland) for Fog-1 and goat anti-human λ-chain F(ab’)_2_ molecules (AbD Serotec or Rockland) for B2 Abs. Human IgA1,κ purified myeloma protein (The Binding Site, Birmingham, UK) or IgA,λ (Jackson ImmunoResearch, Newmarket, UK) were used as negative control test Abs. Complexes were detected using FITC-conjugated F(ab’)_2_ fragments of rabbit anti-goat IgG, F(ab’)_2_-specific Abs (Jackson ImmunoResearch) or FITC-conjugated donkey anti-goat IgG Abs (AbD Serotec).

Levels of fluorescence were determined using a CyAn ADP flow cytometer and Summit v4.3 software (DakoCytomation, Ely, UK) or on a FACScan flow cytometer and LysisII software (Becton Dickinson, Oxford, UK).

### ADCC

Cryopreserved R_1_R_2_ RBCs (50 μL packed cells) were thawed, washed and treated with papain at 37°C for 5 min. They were washed once in PBS, labelled with ^51^Cr at 37°C for 2 h, washed twice and resuspended at 4 × 10^5^ cells/mL in RPMI + 10% FCS. PBMC were isolated by density gradient centrifugation from EDTA-anti-coagulated blood pooled from six normal donors. Cells were washed three times using RPMI containing heparin before adherent cells were removed by incubation in tissue culture flasks at 37°C for 1.5 h in a humidified atmosphere of 5% CO_2_ in air. Non-adherent cells were resuspended in RPMI + 10% human AB serum at 6 × 10^6^ cells/mL.

Fifty microlitres volumes each of Fog-1 Ab dilutions in RPMI, non-adherent mononuclear cells and RBCs were added sequentially to wells of a U-bottomed plate. The plate was centrifuged at 75 × *g* for 3 min and incubated at 37°C overnight in a humidified atmosphere of 5% CO_2_ in air. Samples of 100 μl of the supernatants were counted in a γ-counter. Lysis was expressed as a percentage of the lysis achieved with 1% Triton X-100 after subtraction of spontaneous lysis observed in the absence of test Ab.

### CL assay of monocyte activation by sensitised RBCs

Cryopreserved R_1_R_2_ RBCs were thawed, washed and resuspended in PBS + 0.5% w/v human albumin at 2 × 10^8^ cells/mL. Forty microlitres samples of cells were added to 100 μL volumes of serially diluted Fog-1 Ab in V-bottom well plates and incubated at 37°C for 60 min. The sensitised RBCs were washed three times and resuspended in 200 μL HBSS.

PBMC were isolated by density gradient centrifugation from EDTA-anti-coagulated blood pooled from six normal donors. Cells were washed using PBS + 0.5% human albumin and resuspended in HBSS containing 25% RPMI and 2.5% FCS. Samples of 100 μl were dispensed into wells of a flat-bottomed white opaque 96-well plate and incubated at 37°C for 2 h in a humidified atmosphere of 5% CO_2_ in air. The plates were then placed in a luminometer (Anthos Lucy 1, Labtech International, Ringmer, UK). Hundred microlitres pre-warmed luminol (Sigma) and 20 μL sensitised RBCs were added to each well. The CL response was monitored at 37°C for 60 min, integrated and expressed in Relative Light Units as the mean of the response from duplicate wells.

### Macrophage adhesion and phagocytosis

Mononuclear cells were isolated from individual donors by density gradient centrifugation and added to a six-well plate at 2 × 10^7^ cells/well. The plate was incubated at 37°C for 2 h and non-adherent cells were washed off. Adhered cells were cultured in Macrophage Serum Free Media (Invitrogen) containing recombinant human M-CSF (50 ng/mL, PeproTech, Inc., NJ, USA) for 6 days, with half of the medium being replaced every second day with fresh medium and M-CSF. The cells were then differentiated by 24 h incubation with IFN-γ (50 ng/mL, Sigma) and LPS (10 ng/mL, Sigma). The resultant macrophages stained positive for CD64, CD32 and CD16. RBCs were isolated from an O, RhD-positive donor and incubated with 100 μg/mL Fog-1 Ab for 1 h. The sensitised RBCs were added to macrophages at 1 × 10^6^/well and incubated at 37°C for 1 h before non-adherent RBCs were removed by washing. For each condition, the numbers of RBCs adhered to and phagocytosed by an average of 250 macrophages were determined.

### CL assay of monocyte activation by sensitised platelets

Platelets were obtained from apheresis platelet donors of known HPA-1 genotype 19. To minimise platelet activation, one part platelet-rich plasma isolated from citrated whole blood was diluted sixfold in modified Tyrode's solution with 10% acid citrate dextrose (2.5% w/v tri-sodium citrate, 1.37% w/v citric acid, 2% w/v glucose) and 0.5% BSA. Platelets were centrifuged at 700 × *g* for 10 min, washed three times and resuspended in the same solution at 3 × 10^8^/mL. The preparation was discarded if there were any signs of platelet clumping.

Samples of 66 μL platelet suspension were incubated with anti-HPA-1a Ab or, as negative control, human IgG1 varicella zoster virus Ab (VAZO-5, International Blood Group Reference Laboratory, Bristol, UK) in a U-bottomed 96-well microplate for 30 min at 37°C. Platelets were washed four times and re-suspended in 200 μL HBSS containing 0.5% BSA. Determination of platelet-bound IgG and P-selectin expression was achieved by incubation with RPE-conjugated goat anti-human IgG (Jackson ImmunoResearch Laboratories) or CD62P Abs (AK-6, Serotec). Washed platelets were analysed by flow cytometry (EPICS XL-MCL, Coulter Electronics, Luton, UK). Platelets were identified by particle size using a previously defined region that includes >95% of CD41-positive events and the mean fluorescent intensity of 10 000 events was recorded.

Monocytes were prepared from whole blood as previously described 11. The blood was either pooled from six random donors or from donors typed for the FcγRIIa 131R/H polymorphism. Fifty microlitres sensitised platelet suspension and 50 μL of pre-warmed 4 mM luminol were added to wells containing monocytes in 100 μL HBSS/2% FCS. CL was recorded at 37°C using an Anthos Lucy1 (Labtech International), taking 1 s measurements every 2.35 min for 47 min. The CL response was calculated as the sum of the first seven readings, with each sample tested in duplicate 11. Statistical analysis was performed using the Student's *t*-test for significant differences between treatments.

When examining platelet–monocyte adhesion, monocytes were isolated from donors homozygous for HPA-1b; 5 × 10^6^ HPA-1a/1b platelets were added in 50 μL HBSS/BSA, and platelets and monocytes were incubated together for 30 min prior to addition of HPA-1a Ab and measurement of CL responses as before.

To assess the effect of blocking FcγRI on CL responses, monocytes were incubated for 20 min with 20 μg/mL monomeric murine IgG2a (BRIC163, IBGRL, Bristol, UK). Alternatively, FcγRII was blocked with Fab fragments of mAb IV.3 at 20 μg/mL (Medarex, Minnesota) and monocytes from donors homozygous for the FcγRIIa 131R allotype were used so that blockade of receptor could be confirmed by inhibition of the CL response to a murine IgG_1_ Ab. The CL assay continued with addition of sensitised platelets.

## Abbreviations

ADCC: antibody-dependent cell-mediated cytotoxicity
CL: chemiluminescence or chemiluminescent
HPA: human platelet antigen
P-G1, P-G1/G1Δnab, P-G1Δnab, P-G1Δnac, P-G2: platelets sensitised with B2 IgG that carries the stated constant region

## Supporting Information

As a service to our authors and readers, this journal provides supporting information supplied by the authors. Such materials are peer reviewed and may be re-organized for online delivery, but are not copy-edited or typeset. Technical support issues arising from supporting information (other than missing files) should be addressed to the authors.
